# Collectanea Medica

**Published:** 1812-12

**Authors:** 


### COLLECTANEA MEDICA,

CONSISTING OF

**ANECDOTES, FACTS, EXTRACTS, ILLUSTRATIONS, QUERIES, SUGGESTIONS, &c.**

RELATING TO THE

***History or the Art of Medicine, and the Auxiliary Sciences.***

### EXTRACTS *from* FOREIGN JOURNALS.

### *Analysis of the Urine of the Ostrich.*

MESSRS. Fourcroy and Vauquelin state, that the urine of the ostrich is white, like milk, and usually mixed with more or less of excrement. Its savour is fresh and pungent, like that of a weak solution of nitrate of potass.

They extracted from it uric acid, although it had not before been found in the urine of herbiforous animals. The analysis of the urine presented—1. Uric acid; 2. Sulphate of potass; 3. Sulphate of lime; 4. Muriate of ammonia; 5. An animal matter; 6. An oily substance; 7. Phosphate of lime.

They then examined if the urine of other birds contained uric acid, and succeeded in finding it—1st, in the urine of fowls, uric acid occasioning the white appearance in their excrements; 2d, the dung of turtle-doves contained it; also 3d, the dung of carnivorous birds, especially that of vultures and eagles.

From these experiments, Messrs. Fourcroy and Vauquelin concluded, that it was very probable that the urine of all classes of birds is, with the exception of urea, of the same nature as that of man.—*Journal de Physique, August* 1811.

### *Method of obtaining Artificial Blood, by* M. GRINDEL.

This gentleman has performed a variety of experiments to produce artificial blood. The process which succeeded is as follows:

He mixed and shook, for a considerable time, a dram and a half of white of egg, with five ounces of distilled water; he then added two drams of phosphate of iron, eight grains of carbonate of ammonia, and ten grains of muriate of soda.

This mixture was poured into two cylinders of the double apparatus for analysis, joined one with the gold point in —, the other with the gold point in +, of a galvanic pile of from 160 to 180 lamina of copper and zinc.

This hypothetical composition of the blood, by means of substances, which chemistry, both ancient and modern, has regarded as extracts or products from the blood, and which Messrs. Vauquelin and Fourcroy could not realize, has afforded Dr. Grindel, with the aid of galvanism, a red fluid resembling blood.

In the cylinder of the side +, the fluid presented three distinct layers, the upper one and the lower one of a yellow color, the middle one red. By shaking the mixture briskly, the red color was imparted to the whole fluid. When suffered to rest, it again separated, like blood after bleeding, into a sort of lymph with a coagulated crust, which swam upon the surface. The fluid in the cylinder on the side —, had assumed no color, and resembled chyle.—*Journal Allemand de Berlin.*

### *Analysis of a rose-colored Substance deposited by the Urine in certain Diseases, by* M. Vauquelin.

This matter, which is deposited during certain nervous fevers, has been observed by M. Proust, who has indicated, in the Annales de Chimie, a mode of separating the substances which accompany it, without, however, decidedly ascertaining its nature.

M. Vauquelin has submitted it to several experiments, and found that it is not a simple body, nor even a modification of the urine, but a combination of common uric acid with a coloring matter of a deep red, which is an acid, the properties of which partake more of the nature of vegetable than of animal matter.

The combination of this substance with uric acid seems to be very intimate, for, though much more soluble than this latter, it does not abandon it when it is precipitated from its solution, and the liquor does not retain color when the uric acid is entirely separated. This is also demonstrated by the urine itself, which, after it has deposited its sediment, yields by evaporation only white uric acid.

M. Vauquelin presumes that certain calculi of uric acid which form a rose color, contain this substance; which he proposes, with M. Proust, to term rosacic acid.

He adds the following experiment. After having, by means of a slow heat, evaporated to the consistence of syrup the urine which had formed the rose sediment, he mixed it with with alcohol at 36 degrees, which precipitated from it sulphate of potass, muriate, and phosphate of soda. The alcohol, being filtered, deposited in a few days crystals, which he recognized for the acid phosphate of ammonia.

This fact removes the doubt raised with some plausibility by M. Thenard, upon the nature of the acid which communicates this character to the urine of persons in health; and proves, that, if the acetic acid sometimes exists free in this animal liquor, phosphoric acid may exist there also.—*Annales du Muséum d'Histoire Naturelle, cahiers* 97 & 98.

*Of the Lymph in the Ventricles of the Brain, by* M. HALDAT.

John Hunter, and other authors, had remarked that the lymph in the ventricles of the brain neither coagulated by the action of heat, nor by that of alcohol or of acids. This remarkable distinction, since confirmed by M. Odier, would suffice to indicate a composition different from that of the lymph in the abdominal and thoracic cavities. However, the apparent similarity of these fluids having, till then, induced physiologists to consider them as identical, M. Haldat was determined to submit the fluid to experiments calculated to develope its constituent elements.

Encephalic lymph, the name by which he distinguishes this fluid, is colorless, transparent, nearly inodorous, and has an evidently muriatic savour. It has little viscosity, and does not change by the tincture of mallows; alkalies effect no alteration in it; with the oxy-muriate of mercury, and the muriate of tin, it yields a whitish precipitate; with the nitrate of silver, a crust which turns brown on exposure to air; with decoction of galls, a flocculent deposit of a fawn-color; lastly, with oxalic acid and oxalate of ammonia, a very thin white precipitate.

The whole of these facts indicating a muriatic salt and several animal substances, the author, to isolate them, employed the method used by Dr. Bostock in the analysis of animal fluids, and the other means usually practised. The result of his researches proves that encephalic lymph is composed of the following principles:

| | |
|---|---|
| Water | 96.0 |
| Muriate of soda | 1.5 |
| Albumen | 0.7 |
| Gelatine | 1.0 |
| Mucus | 0.4 |
| Phosphate of soda and of lime, an indeterminate quantity. | |
| Loss or waste | 0.4 |
| | 100.0 |

The

The resemblance between the result of this analysis and that of the fluid of spina bifida, examined by Dr. Bostock, proves the identity of the fluid which moistens the surfaces of the encephalon and those of the spinal canal; and the comparison of the different kinds of fluids employed by nature to prevent inflammation and the coalition of the membranes which line great spaces, seem to divide them into three classes, which M. Haldat has thus arranged:

1. Albuminous lymph of surfaces covered by the serous membranes of the abdomen, of the two pleura, of the pericardium, of the cavity of the tunica vaginalis testis, of the small orifices of the cellular membrane, characterised by the predominance of albumen and soda.

2. The defensive varnish (vernis) of the surfaces covered by mucous membranes, of the primæ viæ, of the air and urinary passages, and of the organs of generation; distinguished by the abundance of animal mucus and muriate of soda.

3. The fluid which moistens the surface of the membranous envelopes of the encephalon and spinal marrow, in which gelatine and muriate of soda prevail.—(*Journal de Physique, Septembre* 1811.)

*Composition of the Anti-syphilitic Tincture of* M. Besnard, *published by order of the King of Bavaria.*

After thirty years of continued success, M. Francis Joseph Besnard, physician to the King of Bavaria, has published the composition of his anti-syphilitic tincture, known and used under his name, through great part of Germany, the kingdom of Bavaria, and in the Bavarian military hospitals. The recipe was published with the authority of the king in the Official Journal of Munich, July 31, 1811; and we now give it as it was reprinted in the Journal de Médecine, published by M. Corvisart, in the Number for September 1811.

*Compositio tincturæ anti-syphiliticæ* Besnardi.

℞ Salis tartari depurati

Aquæ cinnamomi simplicis -- ana ---- ℔j

Opii purissimi -------------------- ℥ij

Aquæ cinnamomi cum vino -------- ℥iv.

Separatim solvantur, dein misceantur invicem, et stent in balneo-mariæ per tres hebdomadas, sæpius agitando:

Gummi arabici electi -------------- ℥ij

Salis alcali volatilis --------------- ℥j

Solve in aquâ cinnamomi simplicis -- ℥vi.

Omnia in se mixta, stent in vase benè clauso per aliquot dies in quiete, dein filtrentur et serventur usui.

*Usus.*—Æger, contracto contagio syphilitico cujusque demum demum sit formæ, ter intrà nychtemerum sumat guttas 24 ex vasculo decocti radicum altheæ refrigerati. Diminuitur ista dosis, dum spatio aliquot dierum symptomata mitigantur ità ut bis, et denique semel, per diem consumpta sufficiat.

Junioribus, et infantibus, dosis, ratione ætatis et virium, administrari debet, et pro vehiculo hisce inservire potest syrupus capillorum veneris vel amygdalinus.

Contra affectiones locales, v. g. ulcera syphilitica, condylomata, rhagades, excrescentias, phymosin, paraphymosin, etc. etc., juvat usui tincturæ interno, ejusdem applicationem quoque externam conjungere, et quidem inservit huic scopo, decoctum radicum altheæ tepidum cum simili tincturæ quantitate uti initio præscripta fuit; sub forma balnei, topici, lotionis, gargarismatis, etc., ad partis affectæ naturam.

Sicubi ulcera pura evadunt, cum merâ tincturâ, ope penicilli ex linteo carpto confecti humectantur, ac nisi hâc methodo plenam sanationem accipiant, lapide infernali quotidie tanguntur, super imposito dein linteo carpto.

Notandum, ulcera hoc modo tractata, etsi pura nec ultrò serpentia, ægrè tamen nec nisi segniter quandoque consolidari et sanari in tali casu viribus prospiciendum dicta et medicaminibus roborantibus.

Bubones inunguntur bis per diem, eâdem tincturâ, et elapso aliquot dierum spatio, teguntur emplastro ex unciâ unâ emplastri diachylon compositi cum gummatibus ex dimidia drachma saponis hispanici confecto, ad perfectam utque dissolutionem vel suppurationem.

Idem emplastrum et contra tumores testiculorum ex gonorrhæâ regressâ ortos, efficax est; nec tamen hic omittendum suspensiorium.

In gonorrhææ stadio inflammatorio convenit partis affectæ balneum suprà memoratum et usus internus decocti altheæ ad libras quatuor cum tincturæ guttis 24 per diem. Fractâ tandem phlogosi tincturâ, modò initio memorato ter ex decocto altheæ hauritur, et simul ter quaterve injectiones fiunt ex aluminis puri drachmâ unâ et unciis quatuor mucilaginis gummi arabici in librâ unâ aquæ distillatæ solutis, in urethram viris, in vaginam fœminis, successu temporis tandem nisi stillare cessat gonorrhæa, huic remedio admiscetur scrupulus extracti Saturni.

Balnea insuper totius corporis ex aquâ tepidâ cum lixivii communis mensurâ vel cinerum clavellatarum portione impregnatâ, victus et potus conveniens ratio, sanatione integrâ multum proficiat.

Reliquis forsan affectionibus, quibus morbi syphilitici non nunquam stipantur medicus lege artis medicitur.

*Anti-*

### *Anti-syphilitic Remedy without Mercury, discovered in Sweden, by* M. ORBEK.

The good effects of this new remedy have been proved in a number of cases in which mercury and other anti-syphilitic substances have been found ineffectual. We give the preparation of it as published in the Swedish Journals.

The principal ingredient is the *chærophyllum sylvestre.* It should be gathered when it begins to be in flower, and the flowers of the plant being dried, an extract is to be made of them. This extract is to be formed into pills of two grains each, and the patient is to take three of them morning and evening for three weeks. During this period he is also to take every day an ounce of *radix chinæ* in decoction, and continue it for six weeks. The decoction is to be made with a sufficient quantity of water, which is to be reduced to the measure of two pints and a half.

During this treatment, the patient's diet is to consist of only two ounces of meat at noon, and as much in the evening, with a small roll of white bread at each meal, as recommended by Winslow.

Having continued the pills of *chærophyllum* for three weeks, without leaving them off, the patient is directed to take in addition, every morning for the same period, pills *ex hydrargyro corrosivo albo*, Ph. Sv. This is the whole process. M. Hufeland, who published this recipe in the *Journal de Médecine-pratique* for August 1811, has confirmed the salutary effects of this remedy, employed in Sweden for some time past, observing, "That this treatment unquestionably has succeeded in different cases, in which mercury and other anti-syphilitic remedies had been employed without any benefit."

---

### *Aphrodisiacs.*

An old Frenchman (*Cossigny*), but a disciple of the new school, has found out that the Chinese are in possession of a new science, the existence of which was not even suspected by the enlightened nations of Europe. As he has the merit of making this wonderful discovery, it is but fair to give it in his own words: "Je pense que nous devrions prendre chez eux (les Chinois) les premiens elemens de la *Spermatologie*, science toute nouvelle pour l'Europe, science qui intéresse l'humanité en genéral, en lui procurant des jouissances qui l'attachent à son existence, en entretenant la santè et la vigeur, en réparant l'abus des exces, en contribuant à l'augmentation de la population. Il seroit digne de la sollicitude des gouvernmens de s'occuper des rescherches qui pourroient donner des connoissances sur une science a

 peine peine soupçonnée des peuples éclairés de l'Europe." He then announces his knowledge in preparing "des petites pastilles qui sont aphrodisiaques et qui convieunent sur tout aux veillards, et à ceux qui ont fait des exces:" and he concludes with the mortifying intelligence that he is not permitted to reveal the important secret, "qui intéresse l'humanité en genéral."

The reputation raised for the Chinese by the Jesuit missionaries; their vaunted skill in the science of medicine; the extraordinary quality of their *tactus eruditus*, by which they discover the causes and symptoms of diseases simply by feeling the pulse without asking a question; the powerful properties of some of their remedies, particularly the *Jaem-saem* (Ginsing), against age and impotence; made the above a speculation of some promise for a native of that country where poverty is science.* The chevalier Cossigny, with his *petites pastilles qui sont aphrodisiaques*, would be a worthy coadjutor to the chevalier d'Husson, with his *eau medicinale*, that cures the gout.

---

### *Eupatorium perfoliatum.*

The late Dr. Guthrie, of St. Petersburgh, was informed, before 1798, by Mr. Frazer, of Chelsea, that in Carolina, Georgia, and East Florida, but more especially in the last-mentioned country, where bilious intermittents are equally frequent and dangerous, the inhabitants use, with wonderful success, as a tonic and febrifuge, the *Eupatorium perfoliatum;* and, as it can be given with equal success during the paroxysms and intermissions, they prefer it to the Peruvian bark.

---

### *Blood-letting in Typhus.*

A large discharge of blood at the very onset of typhus, has, in the hands of some ingenious and judiciously bold practitioners, arrested the disease at once; and, no doubt, by instantly obviating the effect of determination of blood to the brain. Dr. Felix, medical superintendent of the depôt of

---

* "All that at home no more can beg or steal,
Or like a gibbet better than a wheel;
Hiss'd from the stage, or hooted from the court,
Their air, their dress, their politics, import;
Obsequious, artful, voluble, and gay,
On Britain's fond credulity they prey.
No gainful trade their industry can 'scape,
They sing, they dance, clean shoes, or cure a clap:
All sciences a fasting Monsieur knows." LONDON.

French

French prisoners at Stapleton, near Bristol, most happily put this to the test, in the year 1809. During this year, a fever, of the typhoid character, appeared very extensively among the prisoners. The principal remedy employed by this skilful and accurate observer, was depletion, by opening the temporal artery, and abstracting a large quantity of blood. This removed the disease, often without any other remedy of importance, at the end of twenty-four hours, if adopted immediately at the accession of the symptoms. Dr. Felix observed, however, that, from the peculiar circumstances of the situation of the prisoners, there was more inflammatory diathesis at the commencement of this fever, than usually occurs. The circumstances alluded to, rendered the situation of the prisoners necessarily crowded, and generated in their rooms during the night, when all was shut up, a degree of heat fully equal to 100°, which, in the morning, on opening the doors and windows, was suddenly followed by a considerable degree of cold: want of sufficient ventilation, joined with this cause, gave rise to typhus, with organic, chiefly cerebral, congestion.—*Dr. Chisholm.*

---

### *Biographical Memoir of the late Dr. Willan.*

Robert Willan, M.D. F.R.S. and S.A. was born on the 12th of November 1757, at the Hill, near Sedbergh, in Yorkshire, where his father resided, in the enjoyment of extensive medical reputation and practice.* He was educated in the principles of the Society of Friends, and received his scholastic tuition exclusively at Sedbergh; having obtained his classical knowledge at the grammar-school of that place, under the care of the Rev. Dr. Bateman, and his mathematical acquirements, into the higher parts of which he advanced with great success, by the assistance of the celebrated Mr. Dawson. Being early distinguished by his studious disposition and the rapidity of his attainments, he was a favorite pupil with both his tutors, and was advanced by the former into the classes of his seniors, among whom he maintained his station by the excellence of his lessons and exercises. He became ultimately an accomplished classical scholar, and was deemed to excel his master in his critical knowledge of Greek: Mr. Dawson likewise esteemed him one of the most successful students of the mathematics, among the numerous able pupils whom he instructed in that science. The medical profession had long been determined upon as the object

---

* Dr. Robert Willan, senior, graduated at Edinburgh, in 1745, and published an inaugural thesis, "De Qualitatibus Aëris." The Hill is now the residence of his eldest son, Richard Willan, esq.

of of his future pursuit, and he commenced his studies in that line at Edinburgh, in the autumn of 1777. After the usual residence of three years in that university, he received the degree of Doctor in 1780, when he published his inaugural dissertation, "De Jecinoris Inflammatione."

In the autumn of the same year he repaired to the metropolis with the view of obtaining farther medical information, and attended lectures with great assiduity. An arrangement had been made some time previously with Dr. Trotter, a relative, and a physician of some eminence at Darlington, in the county of Durham, but advanced in life; in consequence of which he intended to decline practice in that place in favor of his young friend, as soon as he had completed his studies. When in London, Dr. Willan was introduced to Dr. Fothergill, who, from a just estimation of his talents and acquirements, recommended him to try his fortune in the metropolis, and offered him his assistance. Dr. Fothergill, however, died in the month of December, in that year; and in the commencement of the following year, 1781, the death of Dr. Trotter also occurred; upon which Dr. Willan immediately went to Darlington, where he found two opponents already on the spot: one of these, the late Dr. Rotherham, was afterwards, for some years, Dr. Black's assistant at Edinburgh, and ultimately Professor of Natural Philosophy in St. Andrew's; the other, a gentleman whose name we have not learned, continued to reside there while he lived. Dr. Willan remained at Darlington about a year; during which period he analyzed the sulphureous water at Croft, a village about four miles from that place, and wrote a small treatise respecting its chemical and medicinal qualities, containing also a comparison of its properties with those of the Harrogate waters. This tract was published by Johnson, in 1782, with the title of "Observations on the Sulphur Water at Croft, near Darlington:" and a second edition was printed a few years afterwards.

In the beginning of 1782, not deeming an establishment at Darlington worth contending for, Dr. Willan determined to return to London. The assistance of Dr. Fothergill was now lost to him; but he experienced much active friendship from Mrs. Fothergill, the doctor's surviving sister. His advantage, however, was greatly promoted by the establishment of the Public Dispensary, in Carey-street, which was opened in the commencement of 1783, and was chiefly accomplished by the exertions of some of his friends. He was appointed sole physician to it; and, under his humane and active superintendence, together with that of his able and benevolent colleague, Mr. John Pearson, the surgeon to the institution,

 the the new Dispensary speedily flourished, and became one of the most extensive and respectable establishments of its kind in London. He resided at this time in Bartlett's Buildings, Holborn, with a family connected with the Society of Friends. In March 1785, having passed his examinations before the College of Physicians with great credit, he was admitted a Licentiate of that body; on which occasion he addressed some congratulatory Greek verses to the board of Censors.

About the year 1786, he engaged in the office of teacher, and delivered lectures on the principles and practice of medicine at the Public Dispensary. But his success, we believe, in this undertaking, was inconsiderable. Such attempts, indeed, have seldom proved eminently advantageous, except in connexion with the large hospitals, the reputation accruing from an attendance on these great schools being deemed of almost equal importance to the future success of the student, with the knowledge to be acquired there. At a subsequent period, Dr. Willan received, as pupils at the Dispensary, young physicians who had recently graduated, and who were initiated into actual practice, under his superintendence, among the patients of the institution; a mode of tuition from which they derived much practical knowledge, and were gradually habituated to the responsibility of their professional duties. Upwards of forty physicians, almost all of whom have subsequently attained professional reputation, or now occupy responsible situations, both in this country and abroad, have received the benefit of this instruction.

From the moment when Dr. Willan settled in London, he pursued his professional avocations with an indefatigable industry and attention, of which there perhaps are few examples. He never quitted the metropolis for any consideration of health or pleasure during a period of thirty years. For many years he conducted the medical department of two dispensaries, (having subsequently been favored with an appointment to the Finsbury Dispensary, in addition to that of Carey-street,) during which his unremitting attention to the progress of the diseases which came under his care, is evinced by the prodigious collection of cases, which he has recorded in MS. mostly in a neat Latin style, in which he wrote with great fluency. From this assiduous and patient observation of the phenomena of disease, he, doubtless, acquired that acute diagnostic skill, which is the true characteristic of a sound physician, and which all, who have witnessed his practice, allowed him to have possessed in an eminent degree. This discriminative talent, indeed, has been sufficiently manifested in his great work "on Cutaneous Diseases;" but the delicacy of his tact, which enabled him to to detect, like an accomplished artist, the minute peculiarities in the appearances of these diseases, which escaped the notice of ordinary observers, was very remarkable. During the whole of his career, he was not less assiduously employed in examining the records of medicine; both ancient and modern, than in the actual observation of diseases; of which the learning and critical acumen displayed in his publications, as well as the mass of manuscript collections, which he has left behind, afford abundant proof. His habits of domestic privacy enabled him to dedicate a large portion of time to these researches; and indeed to the unabating ardor with which he applied himself to them, must be attributed that premature injury of his health, which shortened the period of his life.

The rise of medical reputation, unassisted by powerful connections, when all unworthy arts of advancement are disdained, must necessarily be very slow. For a considerable time it has no existence, even in the narrow circle of private friends, whose confidence is placed on older heads; and ultimately, it springs but from the gradual accumulation of individual approbation, as the opportunities of meriting it from time to time occur.

> "Crescit occulto velut arbor ævo

> Fama—."

Dr. Willan's advance to public reputation, and to the consequent emoluments of the profession, was, however, regularly progressive, though slow; and his publications, especially his treatise on the diseases of the skin, upon which his posthumous reputation will principally rest, finally placed his professional character upon high ground. In the spring of 1791, he had the honor of being chosen a Fellow of the Society of Antiquaries. He had been early attached to antiquarian researches, and in his juvenile days had, with considerable industry and accuracy, collected from the Odyssey a history of the manners of the primeval times of Greece. Latterly he communicated some papers to the Society, of which, however, he declined the honor of publication; particularly, a collection of provincial words, and an elaborate essay on the practice of "Lustration by Need-fire," (scarcely extinct in some of the northern counties,) which led him into a curious and extensive research, respecting similar practices in ancient times, and the mythological superstitions connected with them. It was not until the month of February 1809, that he was elected a Fellow of the Royal Society.

The increase of his professional avocations, which had compelled him some time before to resign his office in the

Finsbury

Finsbury Dispensary, led him, in the year 1800, to wish to lessen the fatigue of his duties at the Public Dispensary; and accordingly his friend and pupil, Dr. T. A. Murray, was appointed his colleague in that year. This active and intelligent physician, through whose exertions, aided by the Society for bettering the condition of the Poor, the Fever Institution of the metropolis was established, was unfortunately cut off in February 1802, by the contagion of fever, caught in the infected apartments of the first patients who were admitted into the Institution. Dr. Willan, who had strenuously recommended this establishment, was nominated one of its physicians extraordinary. In December 1803, finding his private practice incompatible with a proper attention to the concerns of the Dispensary, which he had now superintended for the space of nearly twenty-one years, he resigned his office. The governors of the charity, in testimony of their gratitude for his services, and esteem for his character, nominated him Consulting Physician, and made him a governor for life, and likewise presented him with a piece of plate, of the value of fifty guineas, inscribed with a testimonial of their attachment and respect.*

For several years previous to his resignation, Dr. Willan's fame and character had been fully established, and the emoluments derived from his practice very ample. He had, during the preceding course of years, resided successively in Ely Place, Holborn, and in Red Lion Square, in connection with the family before mentioned; and lastly, on his marriage in the spring of 1801, he settled in Bloomsbury Square. He was now not only generally consulted, especially by persons laboring under cutaneous diseases, but was also referred to on all occasions by his professional brethren, as the ultimate appeal on these subjects: for, however generally skilled in every other department of medical practice, his reputation for peculiar knowledge on this point had certainly excluded him, in some measure, from that universal occupation in his profession, to which he was so well entitled.

From his childhood Dr. Willan had been of a delicate con-

* This inscription was written by the late learned and reverend Dr. Matthew Raine, one of the governors of the Dispensary, and was as follows: "Viro integerrimo, artis scientiæque suæ peritissimo, Roberto Willan, M.D. ob felicissimam operam, in morbis egenorum civium sanandis, viginti annos amplius gratuito et strenue navatam, ægrotantium apud Londinenses pauperam Patroni, amico amici, L. L. D. D. D. A.D. 1804, Preside Comite Sandvicense, collatæ pecuniæ Custode Gulielmo Waddington."

stitution; his complexion in early life being pale and feminine, and his form slender. His extremely regular and temperate mode of life, however, had procured him an uninterrupted share of moderate health, and latterly even a certain degree of corpulency of person, though without the appearance of robust strength. In the winter of 1810, some of his friends had remarked a slight shrinking of bulk and change in his complexion: but it was not till the following spring that symptoms of actual disease manifested themselves. Being at this time accidentally called upon to make some bodily exertion in assisting a patient, his respiration became oppressed, and he expectorated some blood. Soon afterwards he suffered two severe attacks of catarrh in immediate succession, which, as he did not desist from his professional avocations out of doors, did not readily subside, and left behind a considerable difficulty of breathing, which rendered the horizontal posture in bed insupportable, with sleeplessness, total loss of appetite, cough, hoarseness, and a very unequal and irregular state of the pulse,—symptoms which seemed to imply an effusion of water into the cavity of the chest, and perhaps into the pericardium. The complexion now became exceedingly sallow, and the tunica conjunctiva of the eyes assumed a yellowish hue. With a view to obtain some respite from professional fatigue, as well as the advantage of a better air, he took a house, in June 1811, at Craven-hill, about a mile from town, on the Uxbridge road, where he spent his time, with the exception of two or three hours in the middle of the day, when he went to Bloomsbury-square, to receive the patients who came thither to consult him. During the months of July and August, partly in consequence of the mildness of the season, and partly of the employment of some active medicines and of the repeated application of blisters, the cough and hoarseness were removed; but the other symptoms underwent little change, and the lower extremities became gradually, but at length severely, anasarcous, from the feet upwards. A sudden unfavorable change of the weather, in September, occasioned a return of the cough and hoarseness, with some expectoration; and the probability of becoming phthisical, under the influence of an English winter, induced him to accede to the strenuous recommendation of some of his friends, and to undertake a voyage to Madeira. He accordingly embarked with his family in the Thames, on the 10th of October; and, after being 53 days on shipboard, detained by foul winds in the Downs and at Portsmouth, he arrived at Madeira on the 1st of December. During this interval, a considerable hardness and tumefaction took place in the abdomen, abdomen, with an effusion of water into that cavity, and he was harrassed by a dysenteric attack. By perseverance in an active course of medicine, however, after his arrival at Funchall, all the symptoms were considerably alleviated; insomuch that, in the month of February, he meditated a return to the south of England in April. But this alleviation was only temporary: his disease was again aggravated; the dropsy, and its concomitant obstruction to the functions, increased; and, with his faculties remaining entire to the last, he expired on the 7th of April, 1812, in the 55th year of his age.

By the death of Dr. Willan, the profession was deprived of one of its bright ornaments, and of its zealous and able improvers; the sick of a humane, disinterested, and discerning, physician; and the world of an estimable and upright man. By his exterior deportment in public, indeed, he was far from rendering justice to his own character. His early education, his studious mode of life, and retiring disposition, prevented that display of his various and extensive knowledge, in mixed society, which delighted the privacy of a small circle of friends, and which was dispensed with much playfulness and simplicity of manner. In all the relations of domestic life, indeed, he was an object of general esteem and attachment. The gentleness and humanity of his disposition were equally conspicuous, in the exercise of his professional duties; in the patient attention with which he listened to the complaints of the sick, whom, in his fullest occupation, he never dismissed from his presence, dissatisfied with the brevity of his inquiries; and in the liberality with which he imparted his assistance, yet refused the remuneration to which he was entitled, when the circumstances of the patient appeared to render it oppressive. In his intercourse with his professional brethren, he was liberal and independent, and extremely tender of giving offence. As a practitioner, as we have already observed, he was a close and faithful observer of diseases, and by the peculiar quickness with which he detected their characteristic appearances, however obscured by complication, he had obtained a copious store of sound experience: yet it has been remarked, that he did not always prescribe with that vigor and decision which so much discriminative talent would have authorised.

As a professional writer, Dr. Willan appeared early, in his contributions to the periodical works. On his arrival in London, he became a member of a private medical society, which held its meetings at a coffee-house, in Cecil-street, and which published two volumes of papers, under the title of "Medical Communications," in 1784 and 1790. In the second of these volumes he published the history of "A remarkable Case of Abstinence," in a hypochondriacal young man, which was uninterrupted for the space of *sixty-one* days, and terminated fatally. We believe that this was the only medical society of which he was ever a member. Several communications from him were also printed in the London Medical Journal, edited between the years 1781 and 1790 by Dr. Simmons. In the 4th volume, p. 421, a short letter of his appears, stating the character of a non-descript *Byssus*, found in the sulphureous waters of Aix, and differing from that which he had discovered in the waters of Croft and Dinsdale. In the 6th volume of the same Journal, he relates a fatal case of obstruction in the bowels, from constriction of the colon near the sigmoid flexure, which prevented any evacuation for upwards of thirty days before death; to which he appended some useful reflections on the diagnostic symptoms of these obstructions, as occurring in the large or in the small intestines. In the 7th volume he describes the cure of a case of chorea, by the cuprum ammoniacum, which, however, he candidly acknowledges, in a subsequent publication,* speedily recurred, and was cured by very different means. In the same volume he relates also a singular termination of abdominal dropsy, by a spontaneous discharge of the fluid per vaginam. And in the 8th volume he has given the detail of seven cases of ague, which were speedily cured by the preparation of arsenic, which had recently been recommended by Dr. Fowler. After the publication of the 11th volume of this Journal, Dr. Simmons commenced a new series, under the title of "Medical Facts and Observations;" in the 3d volume of which a paper of Dr. Willan's appeared, containing a description of several cases of ischuria renalis in children, which was found to be connected with inflammation throughout the mesentery.

In the year 1796, Dr. Willan commenced a series of monthly reports, after the manner of those which Dr. Fothergill had formerly given to the public,† containing a brief account of the state of the weather, and of the prevalent diseases in the metropolis. The practice of a Dispensary, where the diseases of the poor, who are peculiarly exposed to the vicissitudes of the seasons, were observed on a large scale, was particularly favorable for ascertaining the existence of epidemics, and for estimating the state of the public health. By the additional scheme, which Dr. Willan devised, of presenting a

---

* Reports on the Diseases in London, p. 246.

† In the Gentleman's Magaz. vol. xx. *et seq.*

monthly catalogue of the diseases which came under his care, the state of the general health was brought most distinctly under the view. These reports were published in the "Monthly Magazine," which had been recently established, and were continued to the year 1800, when he collected them into a small volume, and published them in 1801, under the title of "Reports on the Diseases in London." This little work is pregnant with important and original medical observations, especially on points of diagnosis, which are the foundation of all rational and successful practice. "Eum verò recte curaturum, quem prima origo causæ non fefellerit."* But, from its unassuming pretensions, and desultory arrangement, it has not been sufficiently known and valued by the profession: it never reached a second edition.

We are unacquainted with the circumstances which originally drew the attention of Dr. Willan to the subject of cutaneous diseases. Most probably his own extreme accuracy made him feel early and acutely the vagueness and confusion of language, which universally prevailed in this department of medicine, while his attendance at a public institution brought many of these diseases constantly under his inspection. So early as 1784 and 1785, that accuracy led him to attend to the *elementary forms* of eruptions, if we may so speak, upon which he saw that a definite nomenclature could alone be founded, and upon which he erected the ingenious system developed in his large work. At that period, in his notes of cases, he has seldom designated eruptions by their ordinary names; but speaks of *papulæ* scorbuticæ, eruptio *papulosa*, &c. In 1786, his notes exhibit still more decisive proofs of the careful attention which he was directing to this subject, in the minute descriptions (accompanied by slight sketches with the pen,) of the forms, magnitude, and progress, of eruptions. The zeal with which he was at the same time investigating the original acceptation of the Greek, Roman, and Arabian, terms, applied to eruptive diseases, is likewise manifested by his copious collections from authors, and by the occasional alterations of the nomenclature, applied in the cases, before he had finally determined on his arrangement. This was probably decided about the year 1789; as in the following year his classification was laid before the Medical Society of London, and honored by the assignment of the Fothergillian gold medal of that year, to the author.

---

* Celsus Præf.

It

It is scarcely necessary to state here, that the ground-work of this arrangement is laid in the external characters, or what we have above called the elementary forms, of eruptions, which are distinguished in the outset by precise definitions; such are pimples, scales, rashes, pustules, vesicles, &c. Upon this plan alone can a perspicuous and intelligible classification be formed, as in other branches of natural history.

It was not till the beginning of 1798, that the first part of this work, including the *Papulous* eruptions, was published, in which, as in the subsequent parts, each variety was represented by a colored engraving. In 1801, the second part, including the *Scaly* diseases of the skin, appeared; in 1805, the third part, comprising only two genera of *Rashes*, viz. measles and scarlet-fever; and, in 1808, the fourth part, comprehending the remainder of the rashes, and the *Bullæ*, or large vesications; the whole containing thirty-three plates, and comprising about half of the classification. Four Orders, characterised by the appearance of Pustules, Vesicles, Tubercles, and Spots, remain unpublished; and, with the exception of the two first genera of pustular diseases, *Porrigo* and *Impetigo*, which have been long delayed from some impediments on the part of the engraver and publisher, the rest of the MS. is probably not at present in a state to meet the public eye.

In the interim, however, from the temporary interest which the investigation of the vaccine question excited, Dr. Willan was induced so far to anticipate the order of *Vesicles*, as to publish, in 1806, a treatise "On Vaccination;" in which he also introduced the subject of chicken-pox (another *vesicular* disease) in consequence of the mistakes, which had been committed, in supposing that this was small-pox, when it occurred after vaccination. Two engravings accompanied this treatise, which exhibited the regular form of the vaccine vesicle, the imperfect vesicle, the three varieties of chicken-pox, and the ordinary porrigo favosa of the face, a disease the origin of which had been falsely attributed to vaccination. Although six years have now elapsed since the publication of this volume, subsequent experience has added no fact of importance to the information which it contains;—another proof of the accuracy of Dr. Willan's observation.

In addition to the writings above-mentioned, which have been committed to the press, Dr. Willan had left some others in an unfinished state. During three or four years previous to his death, he had employed his leisure in a most extensive investigation of the antiquities of medicine, if we may so express ourselves, which he had conducted with his usual felicity of execution. His principal object was the illustration of of four subjects, which are enveloped in no small degree of obscurity, namely, 1. The nature and origin of the epidemic or endemic *Ignis sacer*, which was a frequent cause of much mortality in ancient times, and in the middle ages, and has been confounded with the plague, to which it had no resemblance but in its fatality;—2. The evidence of the prevalence of small-pox, measles, and scarlet fever, not only in the first ages of the Christian era, but at still more ancient periods; of which he has brought together, with great ingenuity, a collection that appears incontrovertibly to establish the affirmative of the question;—3. The history of the Leprosy of the middle ages;—and, 4. That of the Lues venerea. The dissertations relative to the two first-mentioned topics, Dr. Willan had nearly completed, having re-modelled the second, by the aid of a friendly amanuensis, during his residence in Madeira. They contain a very able and original view of the state of disease in the early ages of the world, not founded upon any fanciful explanation of terms, but deduced from a sagacious developement of facts, which have hitherto been concealed under perplexed and mistaken, but sufficiently intelligible, language. He has likewise supported the conclusions, which he has drawn, by evidence collected from sources not usually resorted to in such researches.

Several years ago, Dr. Willan made a collection of observations, in about two thousand patients, with a view to an investigation of medical physiognomy, or temperaments, chiefly in regard to the diseases to which each variety of temperament is peculiarly predisposed, and to the operation of medicines on them respectively. In the prosecution of this inquiry, he procured several drawings (portraits) illustrative of the characteristic marks of the more striking varieties. He arrived at some interesting inferences respecting both the physical and moral constitutions connected with these external characters, but he did not deem the matter sufficiently matured to lay before the public.

In conclusion, we must not omit to mention a juvenile work, published by Dr. Willan, on a theological subject; namely, a Life of Christ, related in the words of the evangelists, of whose details he selected those parts respectively which were most full and explicit; and he illustrated the whole by critical notes and explanations, which were particularly full in regard to the diseases mentioned by those sacred writers. A second edition of this work, with additional illustrations, was published in 1802.—*Edin. Jour.*

---

### Queries for India.

1st. What is the history, use, and preparation, of *Bangue* in in India? What are its synonyms in India, Persia, China, Egypt, and Arabia? Is it prepared only from the wild hemp, or indiscriminately from that and the cultivated? Is it used in conjunction with other narcotics, or only by itself?

2d. Is there any history or tradition of the origin of *Siphilis* in India? Is the Persian Fire precisely the same; or is it no more than a local disease, like gonorrhœa in Europe?

3d. Is the method of supplying *Artificial Noses* from the integument of the forehead a common practice; is it generally successful; and what is the history of its origin?

